# Persistent Pain due to Cement Protrusion After Total Knee Arthroplasty: A Report of Three Cases

**DOI:** 10.1016/j.artd.2024.101334

**Published:** 2024-03-05

**Authors:** Nawaf Alamiri, Justin-Pierre Lorange, Ron Dimentberg

**Affiliations:** a Division of Orthopaedics, Department of Surgery, McGill University, Montreal, QC, Canada; b Division of Orthopaedic Surgery, Department of Surgery, University of Montreal, Montreal, QC, Canada; c Department of Orthopaedic Surgery, Saint-Mary’s Hospital, Montreal, QC, Canada

**Keywords:** Total knee arthroplasty, Chronic pain, Cement protrusion, Arthroscopic cement excision

## Abstract

Chronic lateral knee pain after uneventful total knee arthroplasty can be challenging to manage. We present 3 cases where the pain transiently resolved with injections of local anesthetic. Diagnostic arthroscopy revealed cement protrusion at the lateral femoral bone-prosthesis interface. Passive full knee extension during the curing phase is routine to ensure cement pressurization and optimal bonding. This may enable cement extrusion at the lateral femoral interface and result in persistent pain. Therefore, prevention measures should include thorough visualization of the implant after cementing. Arthroscopic cement excision en bloc is a minimally invasive procedure to treat these patients.

## Introduction

In the first postoperative year, primary total knee arthroplasty (TKA) leads to marked improvements in both pain and function [1]. However, 10%-34% of individuals experience unfavorable pain between 3 months and 5 years after TKA [2]. While pain severity was shown to plateau after 3-6 months [3,4], history of chronic pain, poor mental health, and comorbidities were shown to be risk factors for persistent pain after surgery [5,6]. Intraoperative factors such as implant type and surgery duration played a little role in persistent postoperative pain [5]. Nevertheless, knee pain due to cement extrusion has been documented in patients post-unicompartmental knee arthroplasty [[7], [8], [9], [10]]. Among them, a common explanation was that the shortened incision limits the ability to thoroughly inspect the posterior aspect of the knee. Additionally, the use of an all-polyethylene tibial component further hinders the view and passage of instruments in the posterior compartment. On the other hand, only a few case reports presented patients with persistent pain after TKA caused by cement extrusion, where extraction of the fragments relieved the mechanical symptoms [11,12]. Otani et al. [11] provided the hypothesis that the movement of the knee would result in a shift of the fibular head due to the tension exerted by the biceps femoris, leading to the fibular head coming into contact with the extruded cement.

The aim of this study was to describe 3 cases of patients who had persistent pain after TKA. All of them were relieved after the arthroscopic removal of the protruding cement. Written informed consent was obtained from the 3 patients.

## Case histories

### Case 1

**Figure 1:**
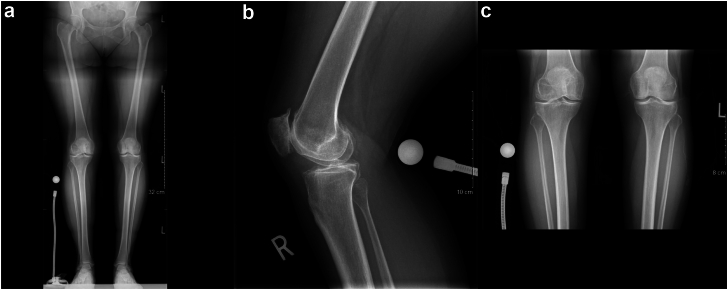
Preoperative images: (a) full-length standing antero-posterior X-rays of both lower extremities; (b) lateral of the right knee; and (c) antero-posterior x-rays of both knees.

**Figure 2:**
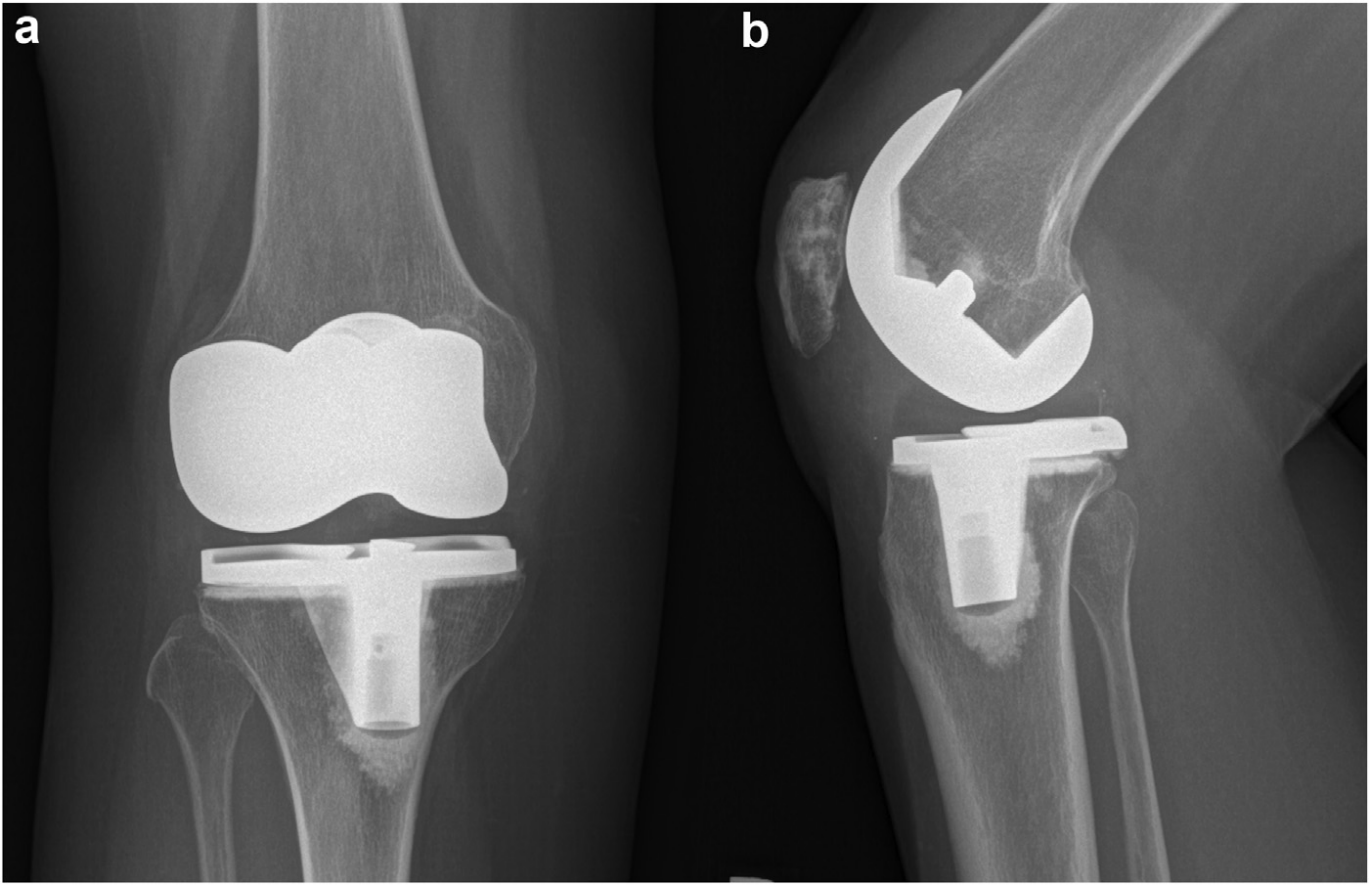
(a) Anterior-posterior and (b) lateral X-rays of the knee after total knee arthroplasty.

**Figure 3:**
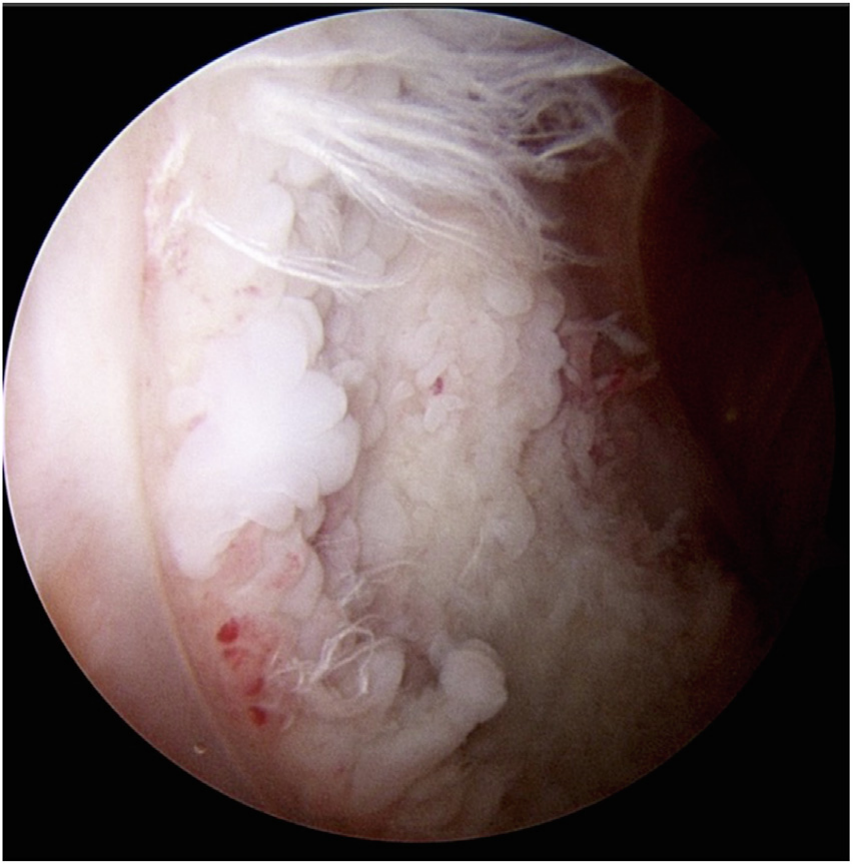
Arthroscopic image of the knee joint showing the synovitis adjacent to the cement (latter is not visualized in the picture).

**Figure 4:**
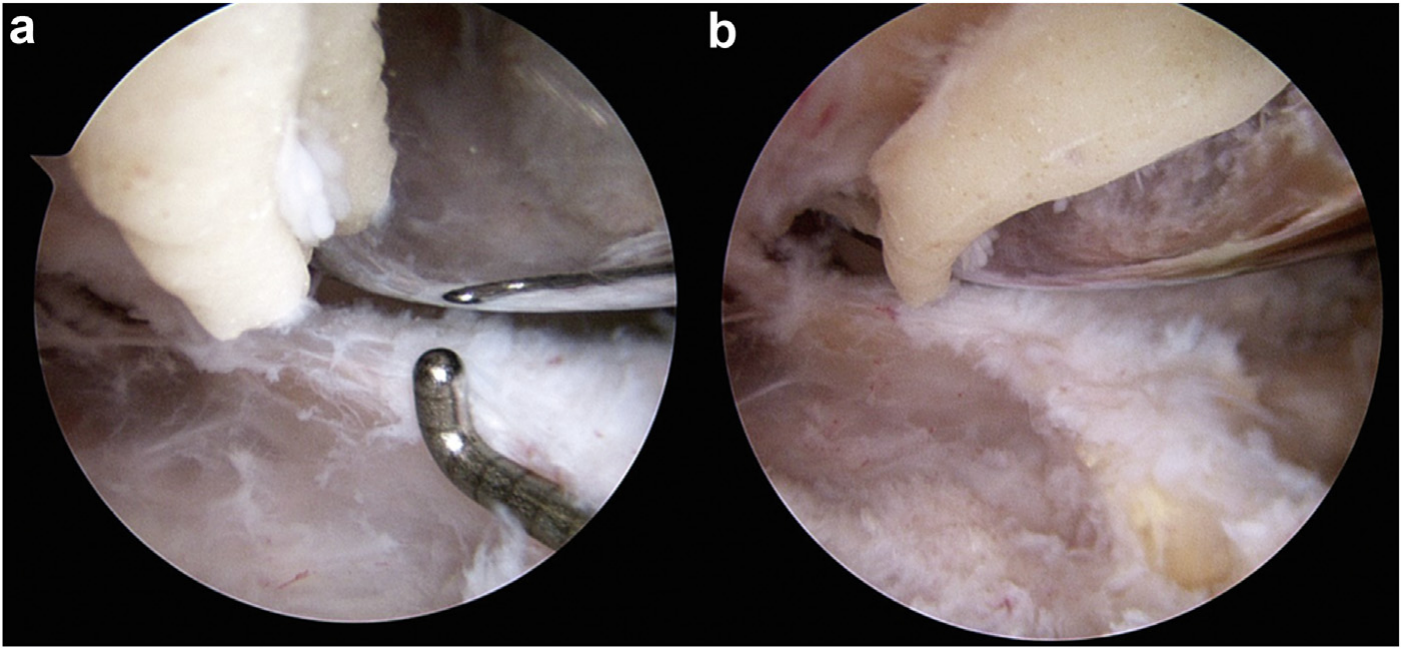
Arthroscopic image of the anterolateral aspect of the knee looking into lateral gutter. (a) A large mass of cement is seen extruding at the lateral bone-prosthesis interface, and (b) the cement mass excised en bloc.

A healthy 53-year-old African Canadian woman underwent a right TKA for severe patellofemoral and medial compartment osteoarthritis (Fig. 1) using a medial parapatellar approach. The femur was addressed first, then the tibia, and no soft tissue release was done. Implants used were Zimmer Persona (Zimmer, Inc., Warsaw, IN) femoral component size 8, tibia size E, a 10 mm vitamin E highly cross-linked polyethylene, and a size 29 patellar component. At the time of surgery, she had a body mass index (BMI) of 27.6 kg/m^2^. Her postoperative course was unremarkable. She was discharged after 3 days with slight knee pain. At 3-month follow-up, she presented with tenderness over the anterolateral knee, near the joint line. She also had a burning sensation over the medial knee and numbness on the sole of her foot. X-rays showed proper placement of the implants (Fig. 2) and physical exam demonstrated full knee range of motion (ROM) (0°-135°), no knee effusion, and good pedal pulses. She was sent for electromyography, blood tests, and repeat X-rays. At 5-month follow-up, the patient had similar symptoms. The electromyography was inconclusive, and the blood tests (C-reactive protein [CRP] and white blood cells) were unremarkable for an infectious process. A small effusion was noted in the right knee. She was advised to repeat the blood tests in 1 month with a follow-up for an injection, if negative. At 6-month follow-up, the patient had an ultrasound-guided lidocaine injection over the area of tenderness, which relieved her symptoms. The patient was booked for an arthroscopy of the right knee for potential lateral cement-loose body. One year post-TKA, she underwent an arthroscopic synovial debridement and excision of cement using 3 arthroscopic ports. A thick synovial plica overriding the medial portion of the patella was removed with the arthroscopic shaver. The medial gutter and suprapatellar pouch were clean. On the lateral retinaculum, there was fibrovascular synovitis (Fig. 3) in addition to a large protuberance of cement on the lateral aspect of the femur component (Fig. 4). The latter was removed using arthroscopic osteotomes. The second postoperative course was unremarkable, and the patient left the hospital the same day. One year after the second surgery, the patient was weight-bearing as tolerated, without pain or neurologic symptoms.

### Case 2

**Figure 5:**
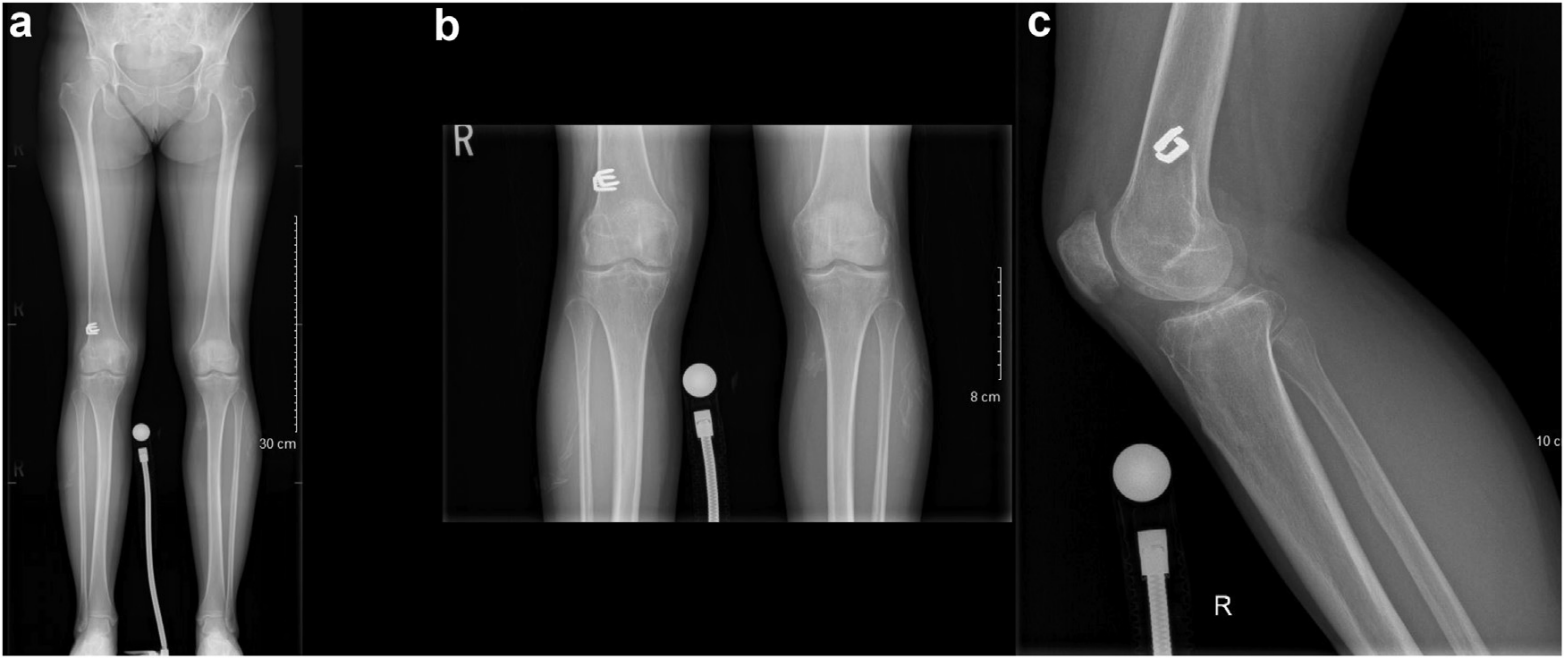
Preoperative images: (a) full-length standing antero-posterior X-rays of both lower extremities; (b) antero-posterior x-rays of both knees; and (c) lateral of the right knee. The anterior cruciate ligament (ACL) staples of the previous ACL reconstruction are visible on all 3 views.

**Figure 6:**
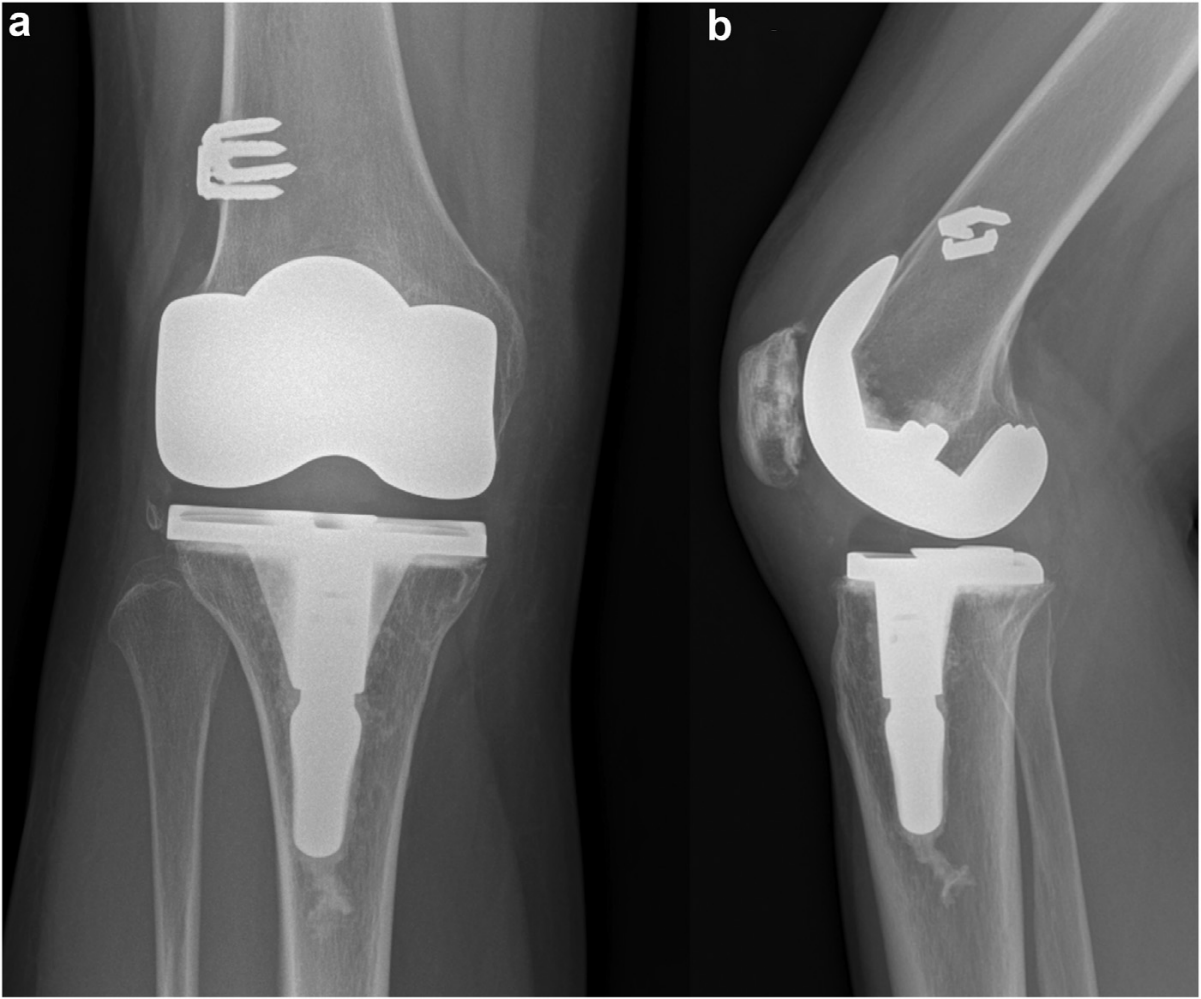
(a) Anterior-posterior and (b) lateral X-rays of the knee after total knee arthroplasty. On both images, the staples of the previous anterior cruciate ligament reconstruction can be seen, and a small fragment of bone is identified, which was not source of pain.

**Figure 7:**
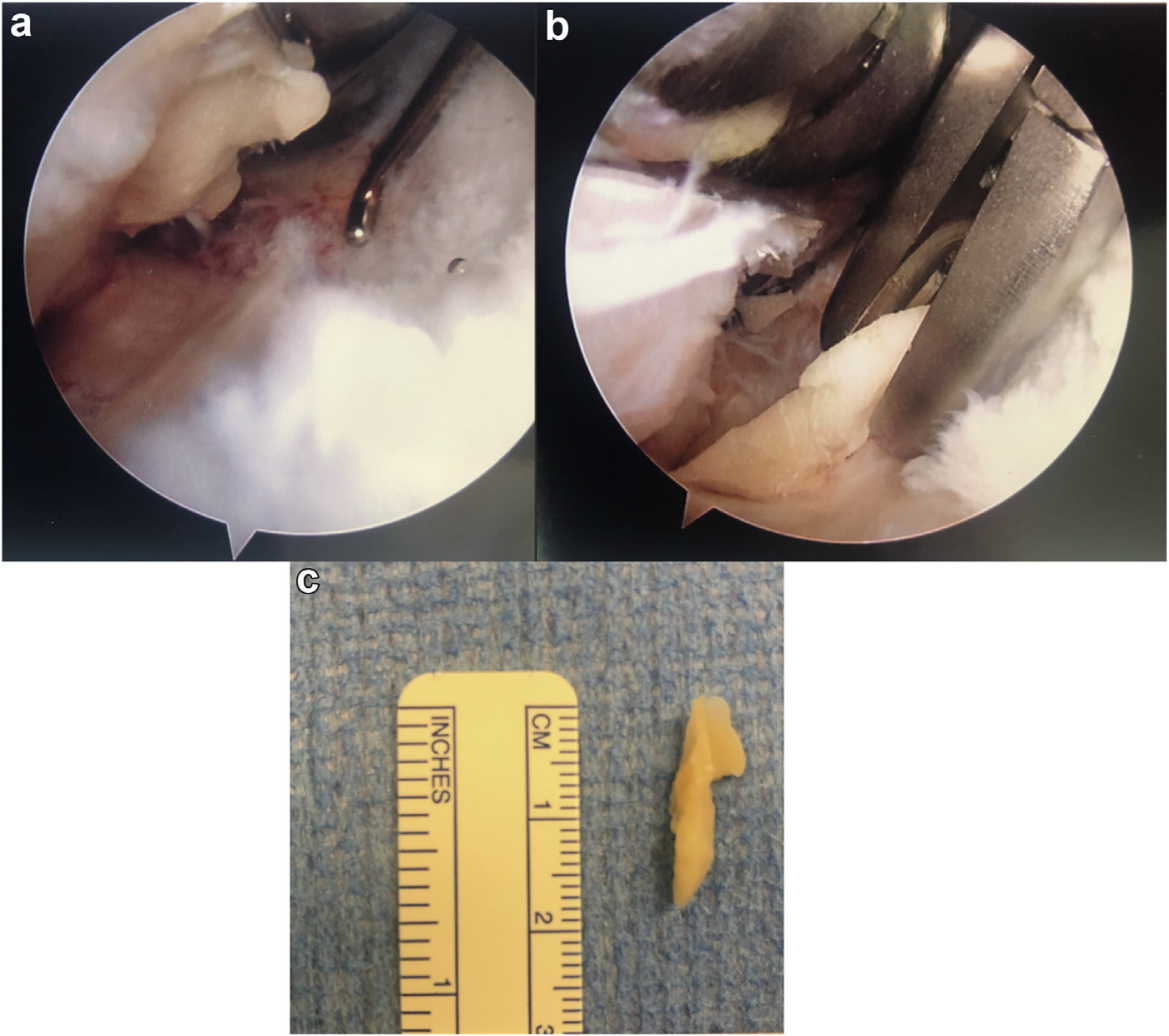
(a) Arthroscope in the superolateral portal looking down the lateral gutter. The probe is shown entering via the anterolatertal portal, with the prosthesis at the top. (b) Extruding cement removed with osteotomes, flush to the prosthesis. The femoral component is seen at the top right of the image. (c) Size of the fragment is assessed using a ruler.

A healthy 55-year-old Caucasian woman with a BMI of 23.0 kg/m^2^ and a history of anterior cruciate ligament reconstruction >10 years earlier was diagnosed with right knee osteoarthritis (Fig. 5). She underwent a right TKA with patella resurfacing using a medial parapatellar approach. The femur was addressed first, then tibia, and no soft tissue release was done. Components used were Zimmer Persona (Zimmer, Inc., Warsaw, IN) Size C tibia, to which a 14 mm diameter and 30-mm stem extension was added, size 6 femur, a 10-mm highly cross-linked Vitamin E cross-linked polyethylene; and a 32-mm patellar component. At 3-month follow-up, the patient had bothersome pain on the anterolateral knee, mostly with activity and stairs. At 10-month follow-up, the patient repeated her X-ray (Fig. 6) and had a ROM of 0°-110°. A small hyperlucent region was identified on the radiographs but was not tender on physical exam. A diagnostic injection on the lateral joint line provided significant relief for a few days. Therefore, she was booked for an arthroscopy of the right knee. One year post-TKA, the patient underwent an arthroscopic lateral gutter synovectomy and excision of protuberant cement. Four arthroscopic portals were made. When looking through the anterolateral portal in the upper portion of the lateral gutter, a large segment of cement (2 cm long × 3.5 mm wide) was seen in the lateral aspect of the knee (Fig. 7). The surgery was uncomplicated. The patient left the hospital the same day. One year after the second surgery, the patient was without pain or neurologic symptoms.

### Case 3

**Figure 8:**
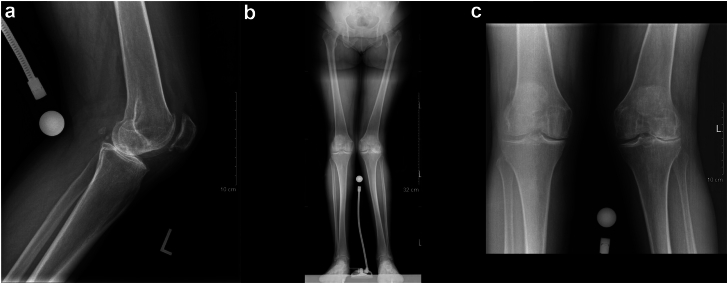
Preoperative X-rays of (a) lateral of the left knee, (b) full-length standing antero-posterior of both lower extremities (c) antero-posterior view of both knees.

**Figure 9:**
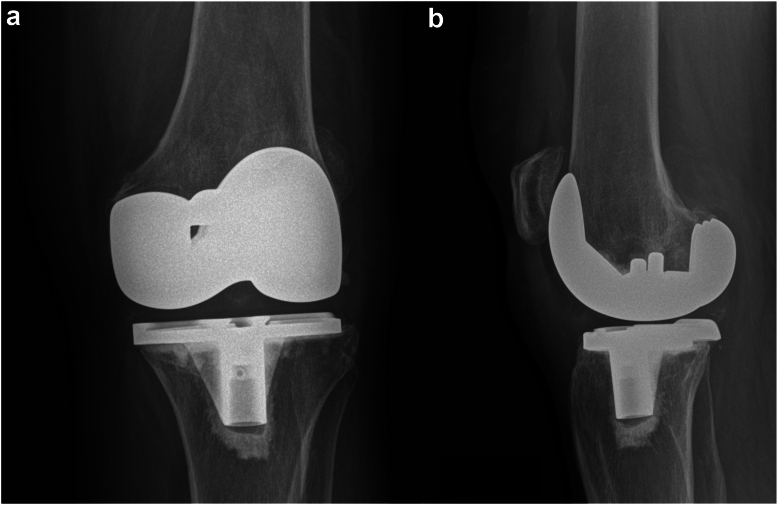
(a) Anterior-posterior and (b) lateral X-rays of the knee after total knee arthroplasty.

**Figure 10:**
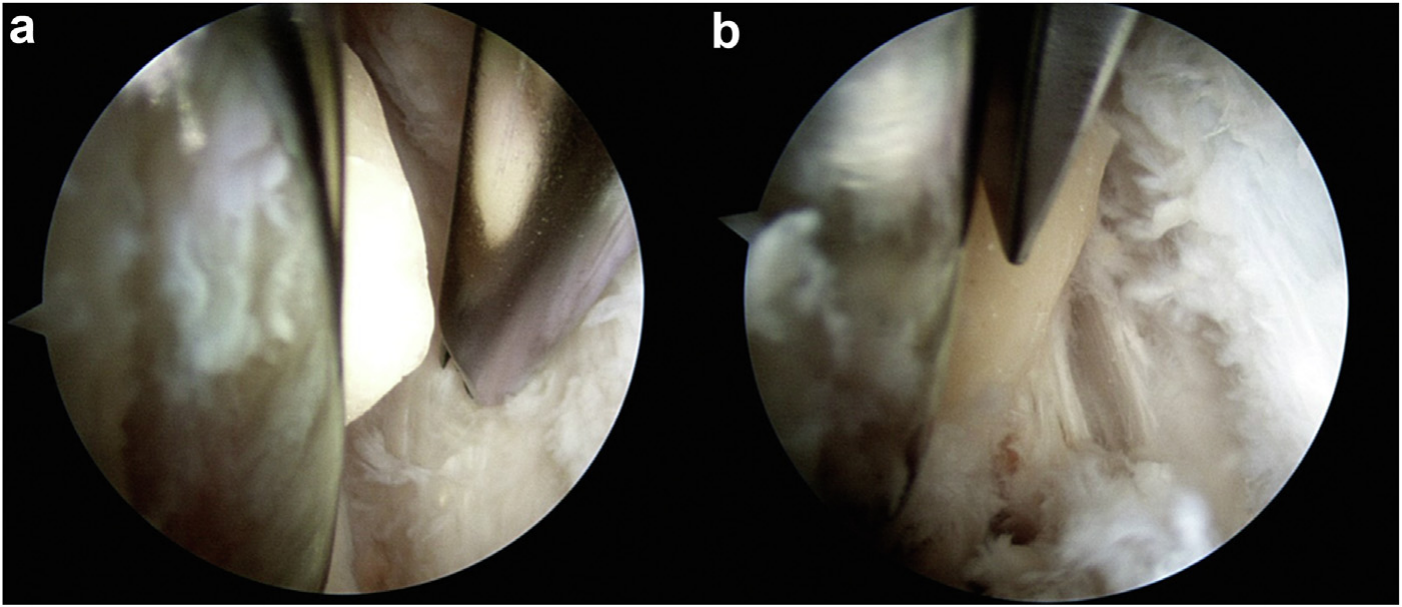
Arthroscopic images looking into lateral gutter show the femoral component (on the left of the image) with cement extruding from the lateral interface. (a) The shaver is seen debriding adjacent synovitis, and subtle striations of the lateral retinaculum is apparent. (b) A standard 1/4" osteotome removes the protruding cement mass flush to bone.

A 69-year-old Caucasian woman with a history of Crohn’s disease and a BMI of 24.8 kg/m^2^ was diagnosed with severe osteoarthritis of the left knee (Fig. 8). She underwent a left TKA using a medial parapatellar approach. The femur was addressed first, then tibia, and no soft tissue release was done. Implants used were Zimmer Persona (Zimmer, Inc., Warsaw, IN) size 8 femoral component, size D tibial component, and a 10-mm vitamin E highly cross-linked polyethylene. At 3-month follow-up, the patient had localized tenderness at the lateral aspect of the knee with good ROM. X-rays were performed, as shown in Figure 9. A mild knee effusion was noted, and synovial fluid was sent for cell count and culture. At 5-month follow-up, the patient had a normal erythrocyte sedimentation rate, CRP, and white blood cells. Synovial fluid culture was negative, despite similar pain as the previous visit. An ultrasound-guided knee injection with cortisone and lidocaine was performed. At 1 year, the pain was still present, despite improvement since the last visit. ROM was 0°-110°. A diagnostic xylocaine injection was performed at the painful site, which provided significant relief 15 minutes after the injection. The patient was booked for an arthroscopy of the left knee. One year post-TKA, the patient underwent an arthroscopic synovectomy with cement extrusion using 3 arthroscopic portals. A large protruding cement mass was removed en bloc in the lateral aspect of the knee (Fig. 10). The surgery was uncomplicated, and the patient left the hospital the same day. After this surgery, the patient had marked varus/valgus instability. Again, investigations included a normal CRP and synovial culture. Therefore, the coronal instability could have been from either under-release or inadvertent over-release of the soft tissue envelope. Nine months after the debridement, the patient underwent a revision left TKA. The intraoperative course and postoperative course were unremarkable. The patient stayed 2 days in the hospital before discharge. At 1 year, the patient was weight-bearing as tolerated, without pain or neurologic symptoms.

## Discussion

During tibial or femoral implant positioning, cement may extrude in the lateral compartment of the knee and go unnoticed. This is most likely due to the pressure applied to the knee when it is in full extension. Herein, we present 3 cases of pain post-TKA that had a common culprit: lateral cement protrusion, which went unnoticed at the time of final knee irrigation.

We found one similar case report on removal of cement protrusion through arthroscopy following TKA [12]. The cement was also located near the lateral femoral condyle. However, they eliminated the protruding cement using an arthroscopic burr. Emphasis was placed on abundant washing of the joint to eliminate all cement particles. For our patients, we used an arthroscopic osteotome to dislodge the cement fragment and a grasper to remove it from the joint space. With this technique, we hypothesize that less cement particles would remain in the joint and potentially cause irritation [12].

The most common cause of early intra-articular pain include infection and instability [13]. After investigation of our patients, our differential diagnosis included aseptic loosening, subcutaneous neuroma [14], and less likely, lateral facet compression due to tight iliotibial band in the unsurfaced portion of the patella [15]. Diagnostic algorithms were published based on the pattern [16] and timing [17] of TKA pain. They suggest that multiple conservative diagnostic procedures and treatments need to be tried prior to considering a second surgery [16,17]. As demonstrated in the above radiographs, cement protrusion is dense and should appear on the follow-up X-rays. While cement extrusion was not mentioned in previously published diagnostic algorithm [16,17], early identification of the protruding cement on X-rays could avoid unnecessary diagnostic tests. For these cases, we found injecting the painful site (lateral knee, around the iliotibial band) useful in our diagnostic workup. These injections can be done with or without ultrasound, depending on the surgeon’s experience and comfort.

Arthroscopy after TKA is a procedure that can be used in the diagnosis and treatment of multiple complications including soft-tissue impingement, arthrofibrosis, or periprosthetic infection [12]. Heaven et al. [18], demonstrated that of all patients who undergo arthroscopy without preoperative diagnosis, 91.1% of them achieve a diagnosis as a result of the surgery. However, this surgery carries inevitable risks such as acute joint infection [19]. When compared to patients who did not undergo arthroscopy, it was calculated that TKA patients who undergo arthroscopy were 3 times more likely to develop a late infection [20]. Another documented complication with arthroscopy post-TKA is related to the “mirror phenomenon.” This is described as the reflection of light on the prosthesis, which makes orientation hard inside the joint space. This heightens the likelihood of generating microabrasions on the surfaces of the components [21]. The use of plastic cannula was suggested in order to reduce this risk [21]. A study by Lovro et al. found that patients undergoing arthroscopy after TKA were 4 times more likely to require revision surgery compared to people who did not, with an overall revision rate of 18.8% [20].

In light of these cases, we believe a prevention approach should be used. Orthopedic surgeon must be aware of this complication and ensure meticulous visualization around the implant before closing the knee after TKA. If significant pain persists after the surgery, a thorough diagnostic process must be followed, ruling out the common reasons for early postoperative pain such as infection and instability. In parallel, careful visualization of the X-rays may reveal cement extrusion. Our clinical intuition leans toward recommending X-ray imaging as a primary diagnostic tool. While other imaging modalities such as computed tomography and magnetic resonance imaging could potentially provide additional information, we have not attempted these methods due to concerns about artifacts. If all other investigations are negative and the diagnosis tends toward cement extrusion, we advise surgeons to attempt conservative management, such as physiotherapy and eventually lidocaine ± cortisone injection. Both investigations and conservative management may span several months. This period allows for observing the natural progression of pain. Once the diagnosis is confirmed and conservative measures prove ineffective, surgery should be considered promptly to prevent unnecessary delays, which will typically occur after a minimum of 6 months post-TKA.

A limitation of this study is differentiating correlation from causation. It is well documented that surgical interventions have an impressive placebo effect [22,23]. All patients in this case series had no pain after the removal of the protruding cement. However, the resolution of the medial knee burning sensation and foot numbness in case 1 remain unexplained. Having data on quality of life would have added an objective parameter to compare the preoperative and postoperative status of each patient. In addition, Figures 6 and 9 show overhang of the lateral compartment, which could be a source of pain. Nonetheless, we believe the arthroscopic debridement alleviated the pressure on the lateral compartment, but a placebo effect cannot be fully excluded.

## Summary

In conclusion, cement extrusion is a rare but disabling complication that may occur despite care to remove protruding cement after TKA. We presented 3 cases of patients post-TKA who experienced persistent pain due to prominent cement in the lateral aspect of the knee. Arthroscopic removal of the cement en bloc provided complete pain relief. During cement pressurization, passive knee extension to confirm joint ROM may result in nonapparent lateral cement extrusion and postoperative lateral knee pain. Specific prevention measures should be followed including thorough visualization of the implant. Mobilizing the patella and lateral retinaculum is suggested to avoid this complication.

## Conflicts of interest

The authors declare there are no conflicts of interest.

For full disclosure statements refer to https://doi.org/10.1016/j.artd.2024.101334.

## Informed patient consent

The author(s) confirm that written informed consent has been obtained from the involved patient(s) or if appropriate from the parent, guardian, power of attorney of the involved patient(s); and, they have given approval for this information to be published in this case report (series).

## CRediT authorship contribution statement

**Nawaf Alamiri:** Writing – review & editing, Conceptualization. **Justin-Pierre Lorange:** Writing – original draft, Software, Formal analysis, Data curation. **Ron Dimentberg:** Writing – review & editing, Supervision, Conceptualization.

## Appendix A Supplementary data

Conflict of Interest Statement for Lorange

Conflict of Interest Statement for Alamiri

Conflict of Interest Statement for Dimentberg
