# Supplementary material for: Persistent Pain due to Cement Protrusion After Total Knee Arthroplasty: A Report of Three Cases
Source: Arthroplast Today. 2024 Mar 5;26:101334. doi: 10.1016/j.artd.2024.101334 (PMC10933466; doi:10.1016/j.artd.2024.101334)
Supplement: Conflict of Interest Statement for Lorange [file mmc1.docx]

# CONFLICT OF INTEREST STATEMENT

***American Association of Hip and Knee Surgeons***

(Adopted from the American Academy of Orthopaedic Surgeons disclosure statement)

The following form **must be filled out completely and submitted by each author (example, 6 authors, 6 forms).**

**All items require a response. If there is no relevant disclosure for a given item, enter "*None*.”**

Manuscript Title

1. Royalties from a company or supplier (The following conflicts were disclosed)

***None***

2. Speakers bureau/paid presentations for a company or supplier (The following conflicts were disclosed)

***None***

3A. Paid employee for a company or supplier (The following conflicts were disclosed)

***None***

3B. Paid consultant for a company or supplier (The following conflicts were disclosed)

***None***

3C. Unpaid consultants for a company or supplier (The following conflicts were disclosed)

***None***

4. Stock or stock options in a company or supplier (The following conflicts were disclosed)

***None***

5. Research support from a company or supplier as a Principal Investigator (The following conflicts were disclosed)

***None***

6. Other financial or material support from a company or supplier (The following conflicts were disclosed)

***None***

7. Royalties, financial or material support from publishers (The following conflicts were disclosed)

***None***

8. Medical/Orthopaedic publications editorial/governing board (The following conflicts were disclosed)

***None***

9. Board member/committee appointments for a society (The following conflicts were disclosed)

***None***

**Each author must sign AND print or type his/her name, date and submit a separate form**

In addition, one BLINDED Conflict of Interest form (no author names used) should be submitted per manuscript with all author disclosures.

Justin-Pierre Lorange
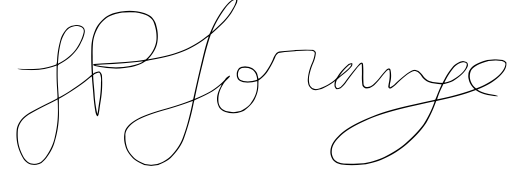
 23/07/2023

Author Name (Print or Type) Author Signature Date
